# Kidney-induced systemic tolerance of heart allografts in mice

**DOI:** 10.1172/jci.insight.139331

**Published:** 2020-09-17

**Authors:** Chao Yang, Jifu Ge, Ivy Rosales, Qing Yuan, Edward Szuter, Ellen Acheampong, Paul S. Russell, Joren C. Madsen, Robert B. Colvin, Alessandro Alessandrini

**Affiliations:** 1 Department of Pediatric Surgery, Guangzhou Women and Children’s Medical Center, Guangzhou Medical University, Guangzhou, China.; 2 Center for Transplantation Sciences, Department of Surgery, and; 3 Department of Pathology, Massachusetts General Hospital and Harvard Medical School, Boston, Massachusetts, USA.; 4 Department of Urology, Shanghai General Hospital, Shanghai Jiao Tong University School of Medicine, Shanghai, China.; 5 Department of Surgery, Boston Children’s Hospital, Boston, Massachusetts, USA.; 6 Organ Transplant Institute, 8th Medical Center, Chinese PLA General Hospital, Beijing, China.; 7 Division of Cardiac Surgery, Department of Surgery, Massachusetts General Hospital and Harvard Medical School, Boston, Massachusetts, USA.

**Keywords:** Transplantation, Organ transplantation

## Abstract

In swine and nonhuman primates, kidney allografts can induce tolerance of heart allografts, leading to their long-term, immunosuppression-free survival. We refer to this phenomenon as kidney-induced cardiac allograft tolerance (KICAT). In this study, we have developed a murine model for KICAT to determine the underlining cellular/molecular mechanisms. Here, we show that spontaneously accepted DBA/2J kidneys in C57BL/6 recipients induce systemic tolerance that results in the long-term acceptance of DBA/2J heart allografts but not third-party cardiac allografts. The state of systemic tolerance of hearts was established 2 weeks after transplantation of the kidney, after which time, the kidney allograft is no longer required. Depletion of Foxp3^+^ T cells from these mice precipitated rejection of the heart allografts, indicating that KICAT is dependent on Treg function. Acceptance of kidney allografts and cotransplanted heart allografts did not require the thymus. In conclusion, these data show that kidney allografts induce systemic, donor-specific tolerance of cardiac allografts via Foxp3 cells, and that tolerance is independent of the thymus and continued presence of the kidney allograft. This experimental system should promote increased understanding of the tolerogenic mechanisms of the kidney.

## Abstract 2

Accepted DBA/2J kidney allografts can confer acceptance of a co-transplanted DBA/2 heart allograft, which would be rejected when transplanted in the absence of the kidney graft.

## Introduction

Establishing donor-specific, immunosuppression-free regimens that result in long-term allograft survival in fully immunocompetent hosts has been a long-standing goal in solid-organ transplantation. Previous studies using rodents, nonhuman primates (NHPs), and swine show that the level of an alloimmune response to grafts is dependent on the relative immunogenicity of the specific organ. Kidney and liver allografts are considered to be “tolerance-prone” organs due to their elicitation of weaker alloimmune responses and relatively easier attainment of tolerance. By contrast, heart and lung allografts are defined as “tolerance-resistant” organs because of higher immunogenic characteristics (1, 2). The reasons for this organ-specific difference in alloresponses are still unclear. In miniature swine (3), different immunosuppressive regimens could induce long-term allograft survival of both class I and full MHC-disparate kidney allografts (4, 5). However, using identical immunosuppression protocols, cardiac allografts were rejected (6, 7). Surprisingly, cotransplantation of both kidney and heart grafts in miniature swine from the same donor resulted in long-term survival of both organs (8, 9). Similar findings were observed in NHPs, where cotransplantation of kidney and heart allografts resulted in tolerance of both organs (M Tonsho and JC Madsen, unpublished observations). This phenomenon, referred to as kidney-induced cardiac allograft tolerance (KICAT), suggests that allokidneys possess organ-specific immunomodulatory capabilities that actively induce systemic tolerance in the recipient that confers unresponsiveness upon cotransplanted cardiac allografts from the same donor.

The overall objective of this study is to identify the mechanisms by which spontaneously accepted murine kidney allografts similarly promote acceptance of heart allografts through the induction of systemic tolerance. It has been well established that, in some MHC-mismatched strain combinations (e.g., DBA/2 to C57BL/6 [B6], B10.D2 to B6AF1, and B10.A to B10.BR), kidney allografts are spontaneously accepted. In contrast, heart allografts from same strain combinations are promptly rejected (10–12). The crucial role of Foxp3^+^ Tregs in spontaneous tolerance of kidneys was confirmed, as evidenced by the rejection of previously accepted renal allografts after specifically depleting Foxp3^+^ cells using Foxp3^DTR^ B6 recipients (13, 14). We hypothesize that the kidney, but not the heart, promotes Foxp3^+^ Treg differentiation in situ and that these cells then circulate to provide systemic tolerance of grafts that share MHC antigens with the kidney.

We have established a model of kidney and heart cotransplantation to further understand the underlining cellular/molecular mechanisms of KICAT. We found that spontaneously accepted kidney allografts can confer systemic tolerance, resulting in the acceptance of donor-specific heart allografts in a Foxp3^+^ Treg–dependent manner and that the maintenance of KICAT is thymic independent. This model will provide us with potentially novel insights on developing strategies that may allow us to induce systemic, donor-specific tolerance of cardiac grafts independent of renal graft cotransplants.

## Results

### Spontaneously accepted kidney allografts induce donor-specific systemic tolerance of cardiac allografts.

MHC fully mismatched DBA/2 kidneys are spontaneously accepted by B6 recipients without immunosuppression, whereas DBA/2 heart and skin grafts are promptly rejected (10). To test if spontaneously accepted DBA/2 kidneys induce systemic tolerance, which, in turn, protected cardiac grafts, we developed a KICAT murine model by implanting cervical heart grafts into recipients of kidney allografts. Bilateral native nephrectomy was performed at the time of kidney transplantation to assess survival and function of the allografted kidney. Cardiac grafts were implanted into the recipients 7 days after kidney transplantation.

**Figure 1:**
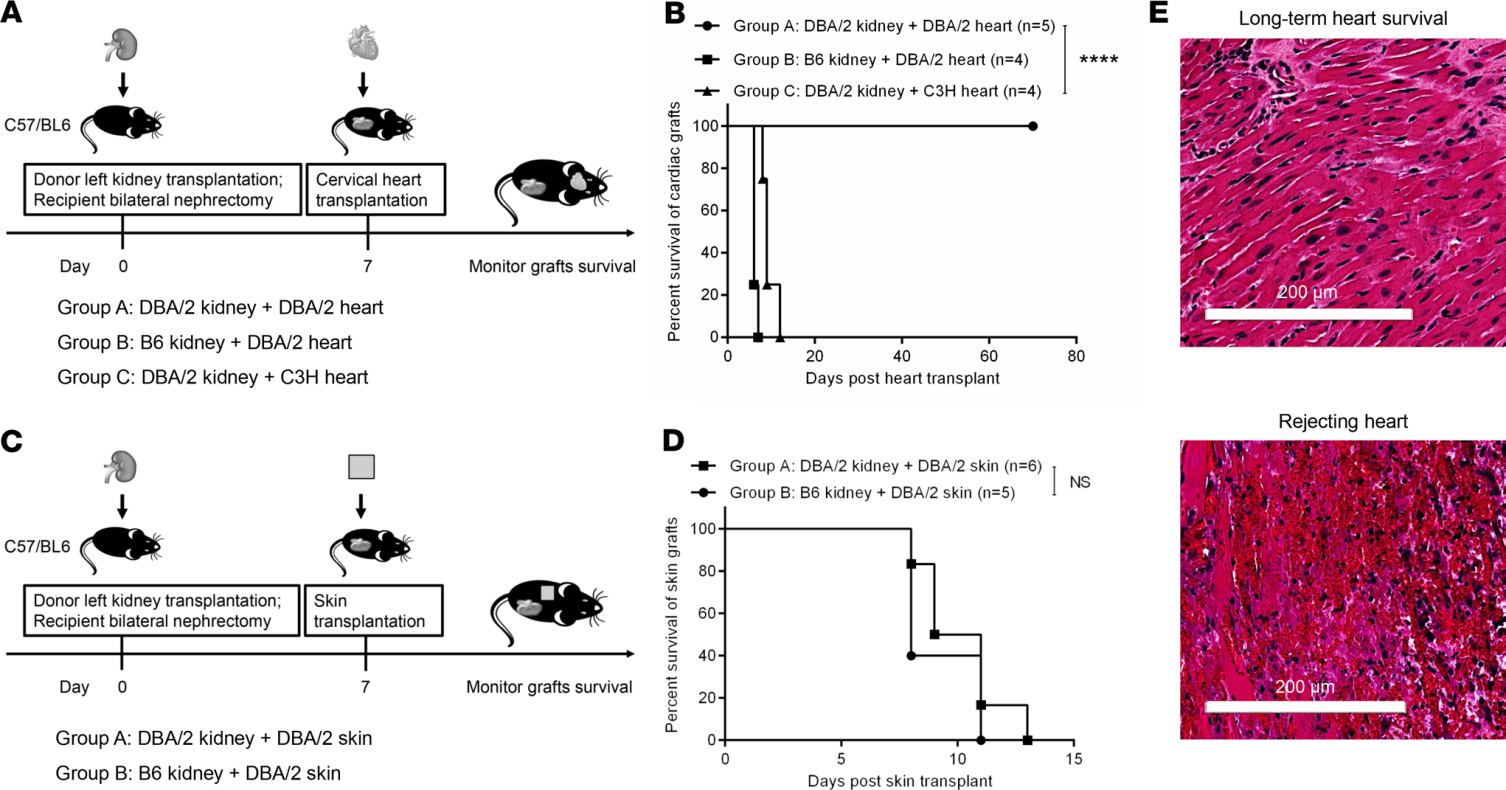
Survival of cardiac and skin grafts in cotransplant recipients. (**A**) Schematic representation of experimental design in kidney and heart cotransplantation. Kidney grafts were first transplanted in recipients that have undergone a bilateral nephrectomy, and cardiac allografts were then cervically implanted into the recipients 7 days after kidney transplant. (**B**) Survival curves of cardiac grafts for each group are shown (group A, DBA/2 kidney + DBA/2 heart, *n* = 5; group B, DBA/2 kidney + DBA/2 heart, *n* = 4; group C, DBA/2 kidney + C3H heart, *n* = 4). Graft survival curves were constructed by the Kaplan-Meier method. ****P < 0.0001, log-rank test. (**C**) Schematic representation of experimental design in kidney and skin cotransplantation. Kidney grafts were first transplanted into recipients that have undergone bilateral nephrectomy, and 7 days later, skin grafts were placed on the recipients. Survival of both grafts was monitored. (**D**) Survival curves of skin grafts in each group are shown (group A, DBA/2 kidney+ DBA/2 skin, *n* = 6; group B, B6 kidney+ DBA/2 skin, *n* = 5). Graft survival curves were constructed by the Kaplan-Meier method (log-rank test). (**E**) Representative images of DBA/2 heart allografts from accepted grafts (group A, top) and rejected grafts (group B, bottom) in **B**. Rejected heart allografts showed marked interstitial hemorrhage and myocyte necrosis. Scale bars: 200 μm.

Cotransplantation of DBA/2 kidneys and DBA/2 hearts into B6 recipients (group A) resulted in long-term acceptance of all cardiac grafts, as assessed by monitoring palpation (100% > day 70, *n* = 5) (KICAT). In contrast, cotransplantation of syngeneic B6 kidneys (group B) failed to prolong the survival of DBA/2 cardiac grafts (100% ≤day 7, *n* = 4). To test whether DBA/2 kidney grafts induce donor-specific immune tolerance, third-party C3H (H-2k) hearts were implanted into B6 mice 1 week after DBA kidney transplantation (group C). As expected, all C3H hearts were rejected acutely by day 12 (*n* = 4) (Figure 1, A and B). Diffuse cardiac myocyte necrosis, edema, and interstitial hemorrhage were present in the rejected hearts (Figure 1E). However, DBA/2 kidneys did not prolong the survival of DBA/2 nonvascularized skin grafts transplanted 7 days after kidney transplants (Figure 1, C and D). These observations show that kidney allografts induce systemic tolerance of MHC-matched cardiac grafts but not skin grafts when transplanted 7 days later.

### Recipient T cell and B cell responses following kidney-induced long-term cardiac allograft survival.

**Figure 2:**
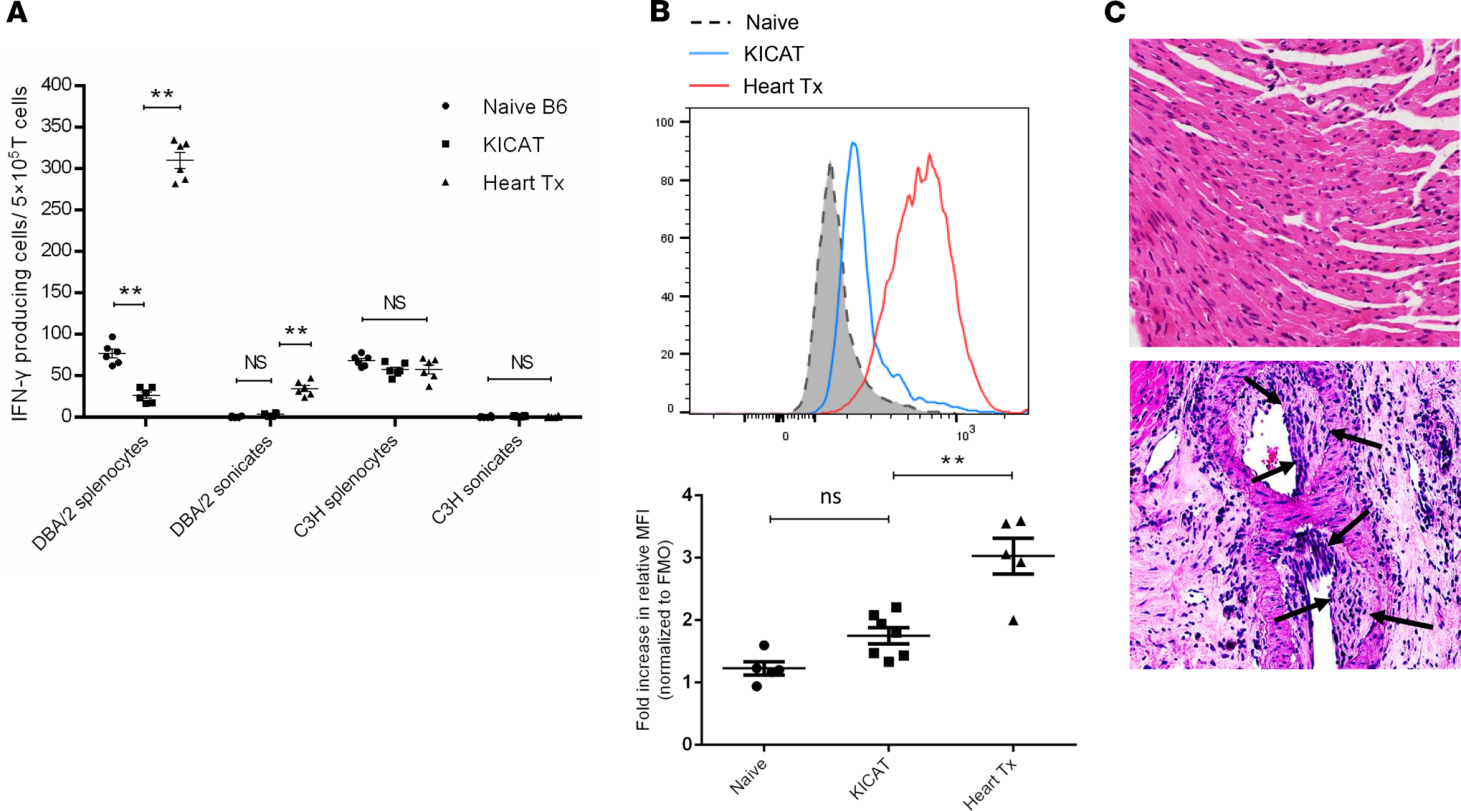
T cell alloresponse and development of CAV in long-term tolerant KICAT recipient mice. (**A**) ELISPOT assay analysis of different experimental groups (*n* = 6 in each group). T cells (responders) enriched from spleens of B6 recipients that received DBA/2 hearts with (KICAT group) or without DBA/2 kidneys (Heart Tx group) as well as from spleens of naive B6 mice were used. Responder cells were stimulated in vitro by syngeneic (B6), donor (DBA/2), or third-party (C3H) splenocytes (direct pathway) and the corresponding sonicates (indirect pathway). Values are shown as mean ± SEM; **P < 0.01, 1-way ANOVA. (**B**) Representative flow cytometric analysis for DSA toward DBA/2 in naive mice (shaded gray) and KICAT recipients (blue) (top). MFI fold change compared with FMO was quantitated (bottom) (*n* = 5 in control groups, *n* = 7 in the KICAT group) (bottom). Values are shown as mean ± SEM; NS, *P* = 0.2575 (naive vs. KICAT) and **P = 0.0012 (positive control vs. Heart Tx), 1-way ANOVA. (**C**) Representative images of DBA/2 heart allografts show myocardium with no signs of rejection (top) and CAV in an aortic branches (bottom) in long-term tolerant KICAT recipients. Mononuclear cells are seen within thickened intima (arrows), associated with reduction in luminal diameter. Scale bars: 200 μm.

The direct and indirect pathway responses of donor-specific T cells in long-term tolerant cotransplant recipient mice (>70 days) were tested using an ELISPOT assay. T cells were enriched from spleens of B6 mice that received DBA/2 hearts, with or without a cotransplanted DBA/2 accepted kidney, as well as from naive B6 mice. The T cells were stimulated in vitro by syngeneic (B6), donor (DBA/2), third-party (C3H) splenocytes (direct pathway) or sonicates from the same strain sources (indirect pathway). When stimulated with both DBA/2 intact splenocytes or sonicates, a significant decrease in IFN-γ production was detected in alloreactive cells from recipients with accepted hearts as compared with recipients with rejecting hearts. In contrast, in vitro alloresponses were similar in both tolerant and rejecting groups toward third-party C3H antigen (Figure 2A). Thus, DBA/2 kidney grafts induce systematic tolerance in a donor-specific manner, with suppression of T cell effector functions, as measured in vitro, by both direct and indirect pathways.

Donor-specific antibodies (DSAs) were assessed by flow cytometry in the long-term accepted DBA/2 to B6 cotransplant recipients. DSA could be detected in the sera of cotransplanted mice when compared with sera from naive recipients, but the difference was statistically insignificant (Figure 2B). These levels were lower than those found in sera from recipients that receive DBA/2 hearts alone. Despite the long-term survival of the heart allografts in the presence of kidney allografts with no signs of acute rejection, cardiac allograft vasculopathy (CAV) was detected in the accepted cardiac allografts (Figure 2C), similar to that reported in other models of tolerance in mice (15–17).

### Kidney allograft removal does not abrogate long-term cardiac allograft survival.

**Figure 3:**
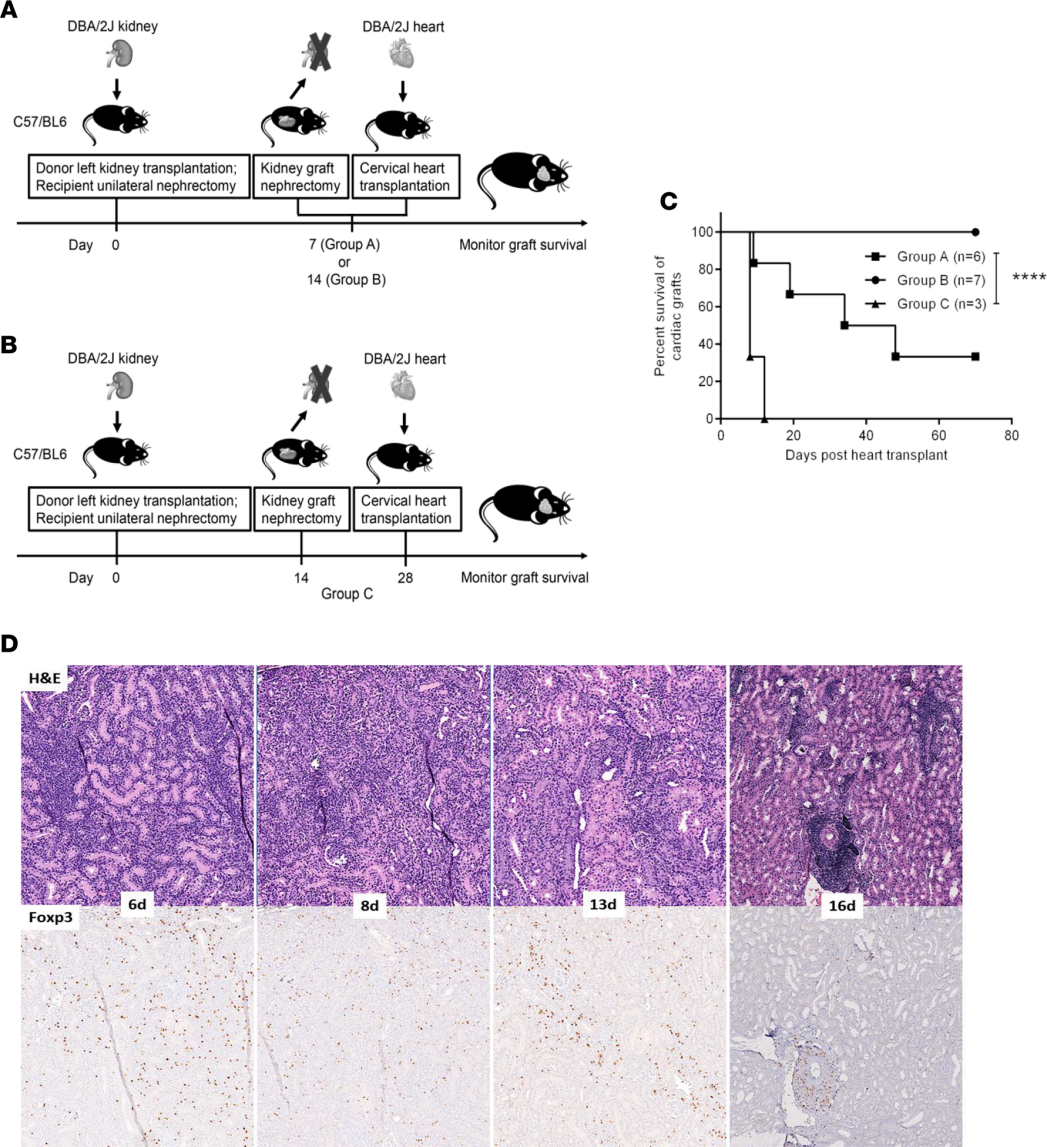
Effect of kidney graftectomy on cardiac graft survival. (**A** and **B**) Schematic representation of experimental design in assessing the influence of kidney graftectomy on cardiac graft survival. DBA/2 kidneys were first transplanted into unilaterally nephrectomized B6 recipients; 7 (group A) or 14 (group B) days later, kidney allografts were removed and recipients received cervical DBA/2 heart transplants. Survival of cardiac grafts was monitored by assessing the cardiac impulse. (**B**) To determine if systemic tolerance is maintained long term after the removal of the kidney allograft, transplantation of heart allografts was delayed 14 days after kidney transplants were removed. Survival of cardiac grafts was similarly monitored by assessing palpations (group C). (**C**) Survival curves of cardiac allografts in each experimental group described in **A** and **B** (group A, kidney allograft removed 7 days after transplant, *n* = 6; group B, kidney allograft removed 14 days after transplant, *n* = 7; group C, kidney allograft removed 14 days after transplant, and heart allograft transplanted 14 days after that, *n* = 3). Graft survival curves were constructed by the Kaplan-Meier method. ****P < 0.0001, log-rank test. (**D**) H&E and Foxp3 staining of DBA/2 kidney allografts isolated on day 6, 8, 13, and 16 after transplantation. TOLS formation is observed within the kidney allograft between 13 and 16 days. Original magnification, ×400.

In our swine model of KICAT, the kidney graft is necessary for the induction phase but not the maintenance phase of cardiac allograft tolerance (18). We sought to understand the influence of coexisting kidney grafts on cardiac allograft tolerance in a murine KICAT model. Kidney allografts were removed 7 days (group A) or 14 days (group B) after transplant, and cervical heart transplants were performed (Figure 3A). In group A, 2 of 6 heart grafts achieved long-term survival (33.3% >70 days, *n* = 6); in group B, all heart grafts demonstrated long-term acceptance after DBA/2 kidney removal (100% >70 days, *n* = 7) (Figure 3C). These data suggest that robust tolerance to the heart allografts induced by kidney transplants was established between 1 and 2 weeks following transplantation. To assess the duration of kidney-induced systemic tolerance, DBA/2 hearts were transplanted 14 days after removal of the allokidney (group C) (Figure 3B). Under these conditions, all cardiac grafts rejected acutely (100% ≤12 days, *n* = 3) (Figure 3C), indicating that kidney-induced systemic tolerance decays in the absence of donor antigen within 2 weeks. These data correlated with the formation of Treg-rich organized lymphoid structures (TOLS) (14, 19) in the kidney allograft, which occurred at between 13 and 16 days after transplantation (Figure 3D).

### Role of the thymus in kidney-induced long-term cardiac allograft survival.

**Figure 4:**
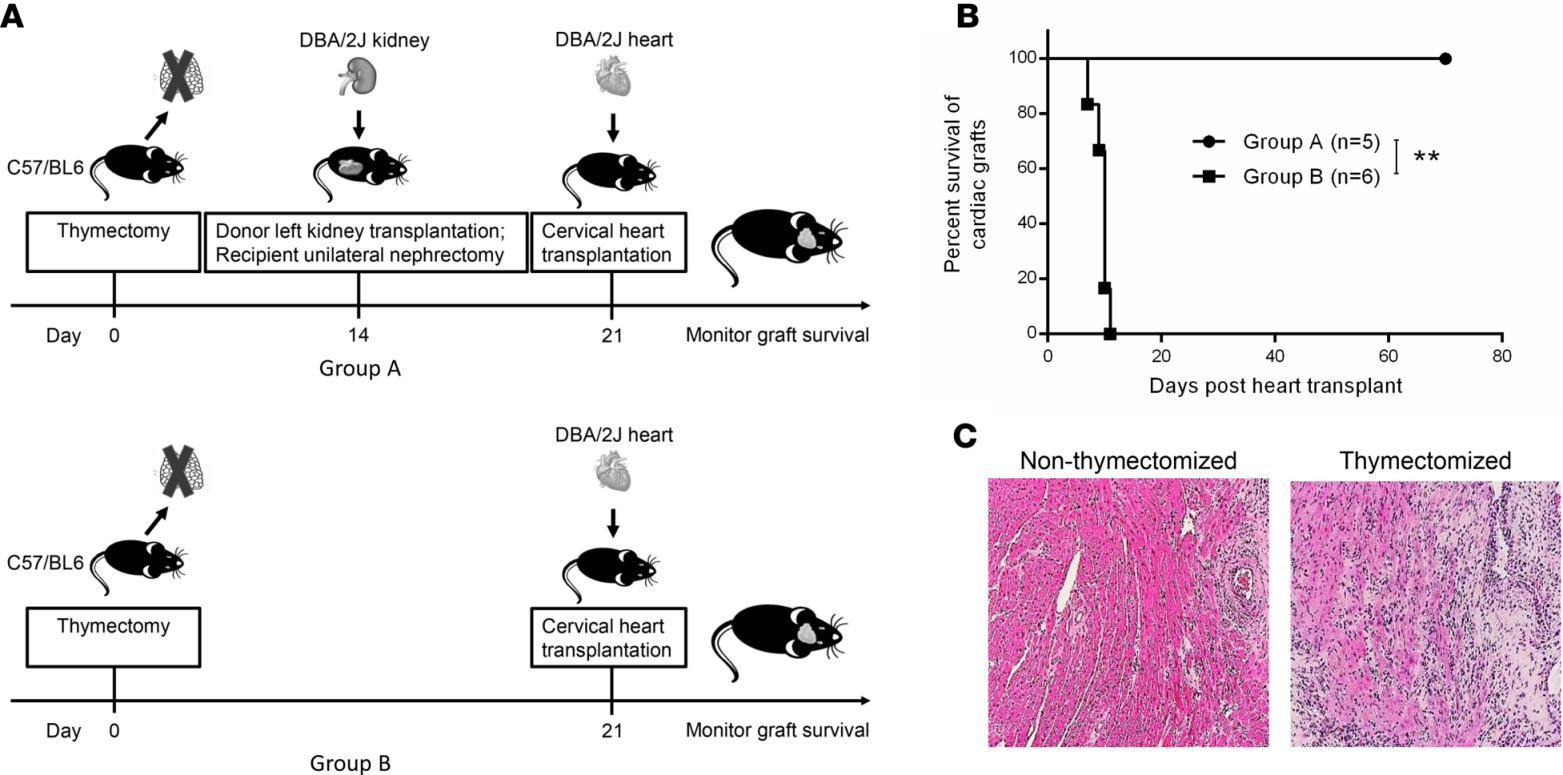
Effect of recipient thymectomy on cardiac graft survival. (**A**) Schematic representation of experimental design in assessing the influence of recipient thymectomy on cardiac graft survival. Thymectomy was first performed on B6 recipients; 14 days later, 1 group (group A) received DBA/2 kidney transplants, followed by cervical heart transplants 7 days later. The control group (group B) consisted of thymectomized B6 recipients that received heart allografts alone without prior kidney transplant. (**B**) Survival curves of cardiac grafts in thymectomized recipients in each group of mice described before (group A, *n* = 5; group B, *n* = 6). Graft survival curves were constructed by the Kaplan-Meier method. **P < 0.01, log-rank test. (**C**) Representative images of DBA/2 heart allografts show mild interstitial infiltrates without myocyte necrosis from nonthymectomized (left) and thymectomized recipients (right) (H&E, original magnification, ×400).

Induction of spontaneous acceptance of DBA/2 kidney allografts by B6 recipients is independent of the thymus, as shown in thymectomized B6 recipients (Figure 4, A and B). To test whether KICAT is dependent on central deletional tolerance mechanisms, thymuses were removed 2 weeks before kidney transplantation and 1 week before cervical heart transplantation. In the absence of the thymus, cotransplanted hearts survived for over 100 days (group A) (Figure 4, A and B). Allohearts transplanted alone in the thymectomized mice (group B) were rejected within 11 days (Figure 4, A and B). The myocardium had a lymphoid infiltrate, but this was not associated with myocyte necrosis typical of active rejection (Figure 4C). Pericardial inflammation with calcification developed in the thymectomized group (probably a surgical artifact) as well as CAV in both thymectomized and nonthymectomized groups. Kidney allografts show well-formed TOLS and no evidence of acute rejection or vasculopathy.

### KICAT is dependent on Tregs.

**Figure 5:**
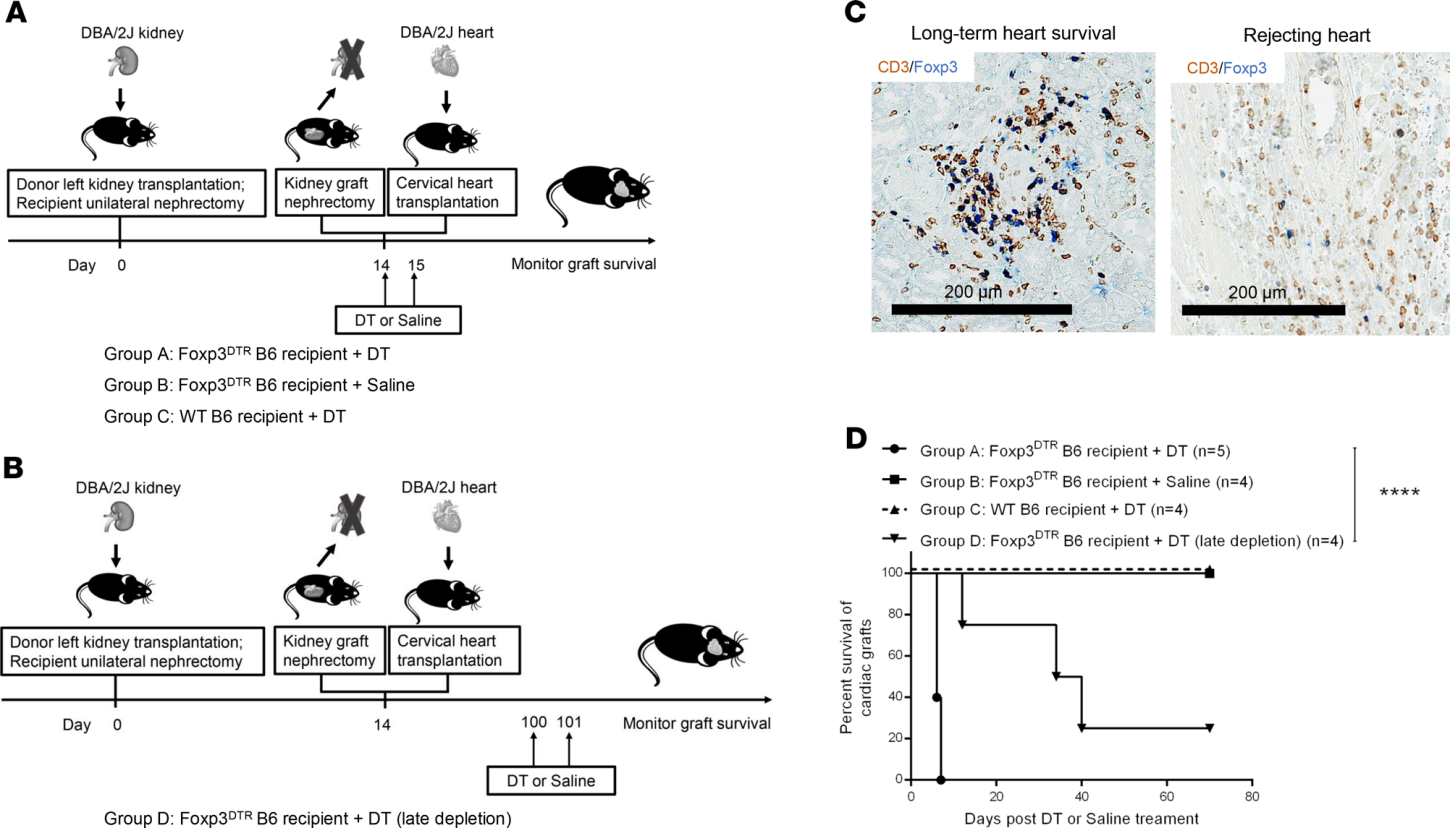
Depletion of Foxp3^+^ cells on cardiac graft survival. (**A**) Schematic representation of the experimental design to assess the effect of early depletion of Foxp3^+^ Tregs on cardiac graft survival in KICAT. DBA/2 kidneys were first transplanted into unilaterally nephrectomized WT or Foxp3^DTR^ B6 recipients; 14 days later, kidney grafts were removed and recipients received cervical DBA/2 heart transplants, combined with either DT or saline treatment, cardiac grafts survival was then monitored. Animals were divided into the following groups: group A (Foxp3^DTR^ B6 recipient + DT); group B (Foxp3^DTR^ B6 recipient + saline); group C (WT B6 recipient + DT). (**B**) Schematic representation of experimental design to assess the effect of late depletion of Foxp3^+^ Tregs on cardiac graft survival in KICAT. DBA/2 kidneys were first transplanted into unilaterally nephrectomized Foxp3^DTR^ B6 recipients; 14 days later, kidney grafts were removed and recipients received cervical DBA/2 heart transplants. One hundred days after transplantation of cardiac allografts, recipient animals received DT treatment (group D), and survival of cardiac allografts was monitored. (**C**) Double immunohistochemical staining of CD3 (brown) and Foxp3 (blue) of DBA/2 heart allografts from long-term tolerant recipients (>day 70), including cotransplant recipients (left), and rejected cardiac grafts (≤day 7) (right). Scale bars: 200 μm. (**D**) Survival curves of cardiac grafts in each experimental group described in **B** and **C** (group A, *n* = 5; group B, *n* = 4; group C, *n* = 4; group D, *n* = 4). Graft survival curves were constructed by the Kaplan-Meier method. ****P < 0.0001, log-rank test.

Spontaneous acceptance of kidney allografts is dependent on Foxp3^+^ Tregs (14). In the murine KICAT model, immunohistochemical analysis of cardiac graft samples from long-term survivors showed an enrichment of Foxp3^+^ Tregs when compared with rejected hearts (Figure 5A). It is likely that peripheral regulatory mechanisms also contributed to KICAT. To test this hypothesis, DBA/2 kidneys were transplanted into unilaterally nephrectomized WT or Foxp3^DTR^ B6 recipients. Fourteen days later, the kidney grafts were removed, and recipients received cervical DBA/2 heart transplants as well as either diphtheria toxin (DT) or saline treatment (Figure 5B) (early depletion). Immunohistochemical analysis shows that heart allografts that survive long-term exhibited greater Foxp3^+^ cell infiltration (Figure 5C). Depletion of Foxp3^+^ cells (group A) resulted in rejection of the cardiac allografts by day 7 (100% ≤7 days, *n* = 5). As a contrast, rejection was not observed in either Foxp3^DTR^ recipients receiving saline (group B) or WT recipients injected with DT (group C) (100% >70 days, *n* = 4) (Figure 5D).

To identify the long-term role of Foxp3^+^ Tregs in the maintenance of immunosuppression-free cardiac allograft survival, recipients were treated with DT 100 days after the heart allografts had been transplanted (group D) (late depletion). Depletion of Tregs in group D recipients resulted in a more gradual rejection, with 3 of 4 grafts ceasing to beat at between day 10 and day 40 after transplantation (Figure 5D). These data suggest that Tregs are essential in both the induction and maintenance phases of murine KICAT. Due to the differential effects of Treg depletion in early versus late DT treatment, early tolerance induction periods are strongly dependent on Tregs, while late stages may be dependent on both Foxp3^+^ cells and non-Foxp3 mechanisms (e.g., deletion).

## Discussion

Our group first showed that, in some strain combinations, mouse renal allografts can survive long term without immunosuppressive treatment (20). Subsequent studies confirmed the spontaneous acceptance of kidney and liver allografts of full MHC incompatibility between specific strains (10, 14, 21–23). In contrast, heart and skin grafts transplanted across identical histoincompatibility barriers are acutely rejected (10–12). Initial studies showed the expression of TGF-β and Foxp3^+^ Tregs in accepted renal allografts (10), and subsequent evidence showed that continued acceptance of kidney allografts was mediated by Foxp3^+^ cells (13, 14). Accepted renal allografts develop characteristic periarterial lymphoid structures containing various immune cell types, which we refer to as TOLS (14). Depletion of Foxp3^+^ cells not only precipitates rejection of the kidney allograft, but also leads to the dissolution of TOLS, suggesting a correlation among formation of TOLS, Tregs, and graft acceptance (14).

The current study demonstrates that kidney allografts induce long-term, immunosuppression-free survival of cotransplanted cardiac allografts within 2 weeks of transplantation. However, kidney allografts did not establish tolerance of nonvascularized skin grafts within the first week. Prior studies using different strain combinations showed that, if kidney allografts were in place for several months, systemic tolerance of skin grafts did occur in about half the recipients (20, 24). The difference in outcome may relate to increased immunogenicity of the skin (including skin-specific antigens) or other mechanisms of tolerance that require a longer time to develop, such as deletion, in recipients of long-term kidney allografts. This emphasizes that heart and skin grafts display different thresholds for tolerance induction. Mechanistic pathways that contribute to the acceptance of cardiac allografts may not be robust enough to confer tolerance of skin allografts in the early stages of systemic tolerance.

Paradoxically, despite the lack of acute rejection of the hearts in KICAT and strong myocyte contraction, CAV in proximal coronary arteries was not prevented by the kidney allograft. In some tolerance studies, such as that described by Turnquist and colleagues, where rapamycin-conditioned DCs were used to induce antigen-specific Tregs in recipients, CAV was not observed in heart allografts (25). In contrast, we have reported consistent CAV in tolerance studies induced by mixed chimerism in mice (17) and in classical murine neonatal tolerance studies (24), wherein CAV limited to proximal coronaries develops in the absence of cellular or humoral rejection. This raises the possibility that the cause of CAV in tolerance settings may be mediated through other mechanisms, such as NK cells, as shown in experimental studies of parent to F1 cardiac grafts (26). We cannot exclude a role for the low levels of DSA detected in the KICAT recipients, even though those levels were statistically insignificant compared with those in naive controls. Furthermore, in separate experiments using an isolated kidney transplant system, where low levels of DSA were detected(14), no evidence of chronic antibody-mediated rejection was observed (26). Further investigations into the causes of CAV in murine KICAT are ongoing.

Tregs were required for the establishment of systemic tolerance in KICAT. Depletion of Foxp3^+^ cells after kidney removal at 2 weeks resulted in acute rejection of the cardiac allografts. However, Tregs were depleted 80 days after hearts were transplanted, and cardiac allograft rejection occurred, but at a much slower rate. This may be due to Foxp3^+^ independent regulatory mechanisms, such as deletion. The continuous presence of the kidney allograft was not required to maintain survival of the cardiac transplant. While removal of the kidney allograft 7 days after transplantation resulted in only a small proportion of cardiac grafts achieving long-term survival, removal of the kidney allograft at 2 weeks resulted in 100% cardiac graft long-term survival. These data correlated with TOLS formation in the kidney allograft, which occurs at between 13 and 16 days after transplantation (Figure 3D). When the kidney allograft was removed at 1 week, only a small proportion of cardiac grafts achieved long-term survival. These data suggest that the induction phase for the establishment of systemic tolerance occurs at between 1 and 2 weeks. After 2 weeks, the presence of the kidney allograft was not required to maintain tolerance of the cardiac allografts, similar to KICAT in the pig (8). We found that systemic tolerance was transient in the absence of continued donor antigen. If heart allografts are transplanted 2 weeks after removal of the kidney graft, hearts are rejected within 12 days. This suggests that the donor antigen may be required to continue induction of suppressor cells, such as Tregs, by tolerogenic APCs, which will be investigated further.

Using thymectomized recipients, 2 main observations were made: (a) spontaneous acceptance of the kidney allograft is not compromised; (b) cotransplanted cardiac allografts exhibited vigorous contractions in both thymectomized and nonthymectomized recipients. This differs from results in the swine KICAT model (8). This suggests that the murine kidney is more able than the pig kidney to substitute for the contribution of the thymus, but it could also be suggestive of differences between spontaneous and nonspontaneous tolerance and would require further studies.

We have shown that Tregs play a role in the establishment of systemic tolerance, similar to their role in the maintenance of spontaneous acceptance. A characteristic feature of spontaneous kidney acceptance is the formation of perivascular aggregates that we have named TOLS with more abundant Tregs (14). These aggregates form by 2 weeks after transplantation, which correlates with our results that the kidney graft must be in place 2 weeks before systemic tolerance is established. Hu and colleagues have shown that Tregs isolated from spontaneously accepted mouse kidney allografts, and not from the spleen of the same recipient, were able to transfer donor-specific tolerance, resulting in longer survival of donor-specific grafts in treated recipients (13). We have also shown that spontaneous acceptance in the mouse is not inhibited in thymectomized recipients, and TOLS still form in renal allografts in these animals. One can surmise that TOLS may play a role in the induction and/or maintenance of donor-specific Tregs, which not only suppress any immune response to the kidney graft itself, but may also migrate into the periphery to induce systemic tolerance and result in tolerance to cardiac allografts. Antigen-presenting cells (APCs), such as immature, conventional DCs and plasmacytoid DCs, found to be present in TOLS (14, 19) and may play a role in the induction and/or maintenance of KICAT. Tolerogenic APCs can induce the transformation of T cells into Tregs, whereas immunogenic APCs lead to development of effector T cells (27). In addition to lymphoid organs, DCs are also found to reside in nonlymphoid organs throughout the body, including kidneys (28, 29). In the field of solid-organ transplantation, resident DCs in donor organs are defined as passenger leukocytes. These cells are generally considered to be initiators of graft rejection through direct allorecognition and/or by releasing exosomes that can trigger an immune response in part through the process of cross-dressing (30–33). We recently published data that show that there is an increase in cross-dressed plasmacytoid DCs within the spontaneously accepted kidney allograft (19). What the functionality of these cells is and the role they play in the induction and maintenance of kidney allograft tolerance is a focus of ongoing research in our laboratory. In certain circumstances, donor passenger DCs can participate in the establishment of transplant tolerance (34). Thomson and colleagues demonstrated that mouse kidney DCs are phenotypically immature and functionally tolerogenic. In vitro assays showed that they were weak in allogeneic T cell proliferative responses and could induce Tregs after repeated stimulation. Furthermore, infusion of these kidney DCs into hosts prolonged allografts survival (35). We found that plasmacytoid DCs, isolated from DBA2/J mice, but not B6 mice, were able to induce Foxp3^+^ Tregs from B6 naive T cells, and this ability of DBA/2 pDCs to induce Tregs correlated with the induction of spontaneous acceptance of kidney allografts (19). One of our findings was that pDCs from various mouse strains had different Treg-inducing potential in in vitro assays (19). These findings suggested that resident donor DBA/2 kidney DCs are likely to be the key players in the initial generation of Tregs by providing direct antigen presentation during the induction phase of kidney-induced tolerance in mice within the first 2 weeks following transplantation of DBA/2 renal allografts into B6 recipients. Direct ablation of these donor DCs using genetic or other approaches would further address their specific role in kidney graft tolerance and KICAT and explain the strain-specific differences in spontaneous tolerance.

To our knowledge this is the first demonstration of KICAT in mice. We showed that spontaneous acceptance of kidney allografts could induce a state of systemic, donor-specific hyporesponsiveness to cardiac allografts and that this is dependent on recipient Tregs but not the thymus. This system will allow identification of cellular and molecular mechanistic pathways of KICAT and the unique tolerogenic pathways of the kidney that may allow development of regimens to benefit clinical heart transplant patients without the need for renal cotransplants.

## Methods

### Mice and treatment

Eight- to twelve-week-old B6 (H-2b), DBA/2J (H-2d), C3H/HeJ (H-2k), and Foxp3^DTR^ mice on the B6 background were used in the experiments. All mice were purchased from Jackson Laboratory. To deplete Foxp3^+^ cells, 50 μg/kg DT (MilliporeSigma) was given intraperitoneally on 2 consecutive days in the Foxp3^DTR^ B6 recipients. All mice were maintained under pathogen-free conditions in filter-top cages throughout the experiments and were cared for by the Center for Comparative Medicine at Massachusetts General Hospital.

### Surgical procedures

#### Kidney transplantation.

The procedure was performed with a modified technique as described previously (20, 36). Briefly, after removal of the recipient left kidney, the aorta and vena cava of the donor kidney were anastomosed end to side to the recipient aorta and vena cava, respectively. The donor ureter was implanted into the recipient bladder and then fixed on the bladder wall. The right recipient kidney was either kept or excised according to the experimental design.

#### Cervical heart transplantation.

Cervical heart transplantation was done by end-to-side anastomosis of donor ascending aorta to recipient carotid artery and end-to-side anastomosis of donor pulmonary artery to recipient external jugular vein. The survival of cardiac grafts was monitored by daily palpation of heart beat. Rejection was defined as the time when cardiac contraction completely stopped by palpation.

#### Skin transplantation.

1 cm × 1 cm full-thickness skin allografts harvested from donor mice were transplanted to the lateral flank of recipients and were held in place by collodion. A gauze dressing was maintained for 7 days after transplant to prevent the graft from being dislodged. Graft survival was monitored by daily visual inspection, and rejection was determined when greater than 80% of the graft became necrotic.

#### Thymectomy.

Via midline cervical incision, 2 thymic lobes were removed by vacuum suction, as described previously (37). The thoracic cavity was then closed immediately after thymectomy to avoid further pneumothorax. Complete thymic removal was confirmed by surgical exploration after subsequent sacrifice of the animals.

#### Kidney allograft removal before cardiac allograft transplantation.

DBA/2 kidneys were transplanted into unilaterally nephrectomized B6 recipients. Kidney allografts were removed at 7 days or 14 days before cervical transplantation of DBA/2 hearts.

### ELISPOT

The MultiScreen filter plates (Millipore) were coated overnight with anti–IFN-γ antibody (BD Pharmingen). A total of 5 × 10^5^ T cells enriched from spleen cell suspensions of the indicated mouse group were placed in each well and were cocultured with intact donor (DBA/2), self (B6), third-party (C3H) splenocytes (direct pathway) or corresponding sonicates (indirect pathway) at 37°C in 5% CO_2_. After 72 hours of incubation, biotinylated anti–IFN-γ antibodies were added overnight and then detected with avidin–horseradish peroxidase and 3-amino-9-ethylcarbazole. Resulting spots representing IFN-γ–producing responders were counted and analyzed by ImmunoSpot (Cellular Technology Limited).

### DSA assay

Sera were collected from indicated groups of mice and then tested for the presence of DSAs using flow cytometry. Briefly, fresh splenocytes from DBA/2 mice were harvested, and red blood cells were lysed. After FcR blocking, 1 × 10^6^ cells in 100 μL PBS were incubated with 4 μl sera collected from indicated mice at 4°C for 30 minutes. After 2 washes, cells were stained with FITC-conjugated rat anti-mouse IgG antibodies (polyclonal, eBioscience) and Percp-cy5.5 rat anti-mouse B220 antibodies (clone RA3-6B2, eBioscience). Sera from naive B6 mice were used as negative controls, while sera from B6 recipients that had rejected DBA/2 cardiac allografts served as positive controls. Mean fluorescence intensity (MFI) values were measured on a B220^–^ cell population and normalized to fluorescence minus one (FMO) control.

### Histology

Kidney and cardiac graft tissues were fixed in 10% formalin and embedded in paraffin. Paraffin sections of 5 μm were further processed for H&E and CD3 (polyclonal, DAKO) and Foxp3 (clone JK-16S, eBioscience) immunohistochemical staining according to the methods described previously by our laboratory (15).

### Statistics

Data were presented as mean ± SEM. Analyses were performed with GraphPad Prism. Graft survival curves were constructed by the Kaplan-Meier method. Statistical significance was assessed by using the log-rank (Mantel-Cox) test. For comparing data among multiple groups, 1-way ANOVA test was used with a Holm-Šidák correction. *P* < 0.05 was considered to be significant.

### Study approval

All procedures were approved by the Institutional Animal Care and Use Committee at Massachusetts General Hospital.

## Author contributions

CY and JG designed and performed experiments, analyzed data, generated figures, and wrote the manuscript. QY performed experiments. IR and EA provided histology analysis. ES and PSR assisted with the editing and writing of the manuscript. AA, RBC, and JCM designed and supervised the study, performed data analysis, and wrote and edited the manuscript.

## Version 1 09/17/2020

Electronic publication
